# First-in-human phase I study to evaluate safety, tolerability, pharmacokinetics, pharmacodynamics, immunogenicity, and antitumor activity of PF-07209960 in patients with advanced or metastatic solid tumors

**DOI:** 10.1016/j.esmoop.2025.104291

**Published:** 2025-02-17

**Authors:** A. Naing, M. McKean, L.S. Rosen, D. Sommerhalder, N.M. Shaik, I.-M. Wang, C. Le Corre, K.A. Kern, N.H. Mishra, S.K. Pal

**Affiliations:** 1 Department of Investigational Cancer Therapeutics, The University of Texas MD Anderson Cancer Center, Houston, USA; 2 Sarah Cannon Research Institute (SCRI), Nashville, USA; 3 UCLA Santa Monica Hematology-Oncology, Santa Monica, USA; 4 NEXT Oncology, San Antonio, USA; 5 Clinical Pharmacology and Translational Sciences, Pfizer Inc., La Jolla, USA; 6 Pfizer Inc., San Diego, USA; 7 Pfizer Inc., San Francisco, USA; 8 Department of Medical Oncology, City of Hope Comprehensive Cancer Center, Duarte, USA

**Keywords:** antibody–cytokine fusion molecule, anti-PD-1–IL-15 fusion, phase I study, advanced or metastatic solid tumors, anti-drug antibodies

## Abstract

**Background:**

PF-07209960 is an antibody–cytokine fusion molecule that consists of a single potency-reduced interleukin-15 (IL-15) mutein and a bivalent high-affinity anti-programmed cell death protein 1 (PD-1) full-length IgG. This phase I study (NCT04628780) evaluated the safety, tolerability, pharmacokinetics (PK), pharmacodynamics, and potential clinical benefits of PF-07209960 in patients with selected locally advanced or metastatic solid tumors for whom no standard therapy was available.

**Materials and methods:**

Escalating doses (1-30 mg) of PF-07209960 were administered subcutaneously once every 2 weeks in 28-day cycles. The primary endpoints included dose-limiting toxicities (DLTs), adverse events (AEs), and laboratory abnormalities. The secondary endpoints included PK, anti-drug antibodies (ADA) and neutralizing antibodies (NAb) against PF-07209960, and tumor response assessed using RECIST version 1.1.

**Results:**

Thirty-seven patients received treatment with PF-07209960 (1-, 3-, and 10-mg groups, *n* = 4 each; 15 mg, *n* = 3; 20 mg, *n* = 16; 30 mg, *n* = 6). The median age was 59.0 years (range 31-88 years). Six (22.2%) patients had DLTs. The most frequently reported treatment-related AEs (TRAEs) (≥50%) were general disorders and administration site condition [21 (56.8%)] and skin and subcutaneous tissue disorders [20 (54.1%)]. The most frequently reported grade ≥3 TRAE was anemia [5 (13.5%)]. Two patients with microsatellite-stable colorectal cancer had confirmed partial response, one each from the PF-07209960 20-mg and 30-mg cohorts, with a duration of response of 9.5 and 3 months, respectively. The rate of ADA was 93.9% (31/33), of which 63.6% (21/33) was treatment induced and 30.3% (10/33) was treatment boosted.

**Conclusion:**

PF-07209960 was generally manageable, with potential antitumor activity in some patients.

## Highlights

• PF-07209960 is a fusion of a single potency-reduced IL-15 mutein and a bivalent high-affinity anti-PD-1 full-length IgG.
• Thirty-seven patients with advanced/metastatic solid tumors received PF-07209960 (1-30 mg); the most frequent G ≥3 TRAE was anemia.
• Two patients with microsatellite-stable colorectal cancer had partial response (duration of response 9.5 and 3 months).
• The rate of ADA development was 93.9% (31/33): 63.6% (*n* = 21) was treatment induced and 30.3% (*n* = 10) was treatment boosted.
• PF-07209960 was generally manageable, with potential antitumor activity in some patients.

## Introduction

Programmed cell death protein 1 (PD-1) is an immune checkpoint expressed primarily on T cells inhibiting T-cell activation. Antibodies blocking PD-1 or its ligand programmed death-ligand 1 (PD-L1) are available as the standard of care (SoC) to treat various types of cancer,^1^, ^2^, ^3^, ^4^, ^5^ but some patients treated with anti-PD-1/PD-L1 therapy do not respond to treatment.

Interleukin-15 (IL-15) and IL-2 bind to IL-15 receptors (IL-2Rβ/γ) to induce immune cell proliferation and survival as well as enhance cytotoxic and cytokine-secreting effector functions of lymphocytes.^6^, ^7^, ^8^, ^9^, ^10^, ^11^ Recently, IL-2 (aldesleukin) was approved for use in metastatic renal cell carcinoma (RCC) and melanoma,^12^ whereas in a clinical trial evaluating recombinant human IL-15, dose-limiting toxicities (DLTs) stopped dose escalation without any objective responses.^13^ The preferential stimulation and resulting uptake of IL-2/IL-15 therapeutic agents by the peripheral blood natural killer (NK) cells may limit the exposure needed to activate CD8+ T cells within the tumor environment,^13^^,^^14^ as intratumoral CD8+ T cells have been associated with efficacy.^15^^,^^16^ However, activation of NK cells mediates toxicity in animal models, and likely in humans, based on the similarities in toxicities observed in the clinic and in animals.^17^

Various approaches have been explored to improve upon IL-2/15 efficacy, pharmacokinetics, and toxicity, such as reducing binding to IL-2Rα^18^^,^^19^ and targeting IL-2 to tumors through fusion proteins.^20^, ^21^, ^22^ Combinations of anti-PD-1 antibodies and IL2/15-based molecules showed promising antitumor activity beyond the efficacy observed with these therapies as monotherapies,^19^^,^^23^ suggesting that resistance mechanisms to anti-PD-1/PD-L1 might be potentially overcome by cytokines.

PF-07209960 is an antibody–cytokine fusion molecule that consists of a single potency-reduced IL-15 mutein and a bivalent high-affinity anti-PD-1 full-length IgG. It was designed to deliver PD-1-mediated avidity-driven IL-2/15 receptor stimulation preferentially to PD-1-positive CD8+ T cells, which are enriched in tumors and can mediate antitumor activity, while reducing the natural preference of IL-15 for a majority of PD-1-negative NK cells, which may mediate toxicity. The exposure of the IL-15 mutein has been extended by fusing it to an antibody, which also reduces its potency to prevent systemic activation of PD-1-negative lymphocytes.^24^ Preclinical data showed that the stimulatory activity of PD-1-targeted IL-15 mutein was enhanced in tumor-infiltrating lymphocytes (TILs) preferentially over peripheral lymphocytes, leading to greater antitumor activity compared with anti-PD-1 and IL-15 agonist alone or in combination even in immunologically cold tumors that are not typically responsive to anti-PD-1 therapy.^24^^,^^25^ Moreover, PF-07209960 was shown to stimulate proliferation of colorectal cancer (CRC) PD-1+ CD8+ TILs preferentially over other lymphocytes, suggesting there may be a potential for activity even in a tumor type considered traditionally resistant to immune-based therapies.^24^^,^^25^

The purpose of this first-in-human phase I study is to evaluate the safety, tolerability, pharmacokinetics (PK), pharmacodynamics, and potential clinical benefits of PF-07209960. Tumors resistant to prior anti-PD-1/PD-L1 therapy, such as non-small-cell lung cancer (NSCLC), squamous cell head and neck carcinoma (SCCHN), RCC, and urothelial carcinoma (UC), and without available anti-PD-1/PD-L1 SoC therapy, such as ovarian cancer (OvCa) and microsatellite-stable CRC were included.

## Materials and methods

### Study design

This is a phase I, open-label, multicenter, multiple-dose, dose-escalation, safety, PK, and pharmacodynamics study (NCT04628780) of PF-07209960 in adult patients with selected locally advanced or metastatic solid tumors (anti-PD-1/PD-L1-resistant NSCLC, SCCHN, RCC, UC; or anti-PD-1-naïve OvCa and microsatellite-stable CRC) for whom no standard therapy was available or appropriate, or who had refused standard therapy (Supplementary Figure S1, available at https://doi.org/10.1016/j.esmoop.2025.104291). Escalating doses (1-30 mg) of PF-07209960 were administered as subcutaneous injection once every 2 weeks in 28-day cycles. A Bayesian logistic regression model guided by the escalation with overdose control (EWOC) principle^26^ was used to guide dose escalation and determine the maximum tolerated dose (MTD)/recommended phase II dose (RP2D). Treatment with PF-07209960 continued until disease progression, consent withdrawal, or occurrence of unacceptable toxicity*,* whichever occurred first; treatment beyond disease progression was based on individual benefit/risk assessments.

This study was conducted in accordance with the Declaration of Helsinki and followed all applicable ethical guidelines, laws, and regulations. The protocol was approved by the ethics committee or the institutional review board. All patients provided informed consent before undergoing study-specific procedures.

Study was closed due to sponsor decision and the dose-expansion part was not initiated.

### Patients

#### Inclusion criteria

Eligible patients were adults (aged ≥18 years) with histological or cytological diagnosis of locally advanced/metastatic NSCLC, SCCHN, RCC, UC, OvCa, or microsatellite-stable CRC who had progressed or relapsed after ≥1 prior line of therapy for recurrent or metastatic disease, including either SoC or investigational therapies, or been intolerant to SoC. Patients with NSCLC, SCCHN, RCC, or UC must have had radiographic progression or relapse from prior anti-PD-1/PD-L1 treatment either as monotherapy or in a combination regimen, with prior last anti-PD-1 dose ≥90 days. Patients with NSCLC must not have known *ALK* or *ROS1* translocations, *MET* exon 14 skipping, *RET* rearrangements, or *EGFR* mutations. Patients with OvCa or microsatellite-stable CRC must not have had been treated with anti-PD-1/PD-L1 therapy. All patients must have had radiographic progression per the Response Evaluation Criteria in Solid Tumors (RECIST) version 1.1; an Eastern Cooperative Oncology Group (ECOG) performance status score of 0-2; and adequate cardiac, hematologic, liver, renal, and coagulation functions.

#### Key exclusion criteria

Patients were excluded from the trial if they had symptomatic brain or leptomeningeal metastases requiring steroids; any other active malignancy (excluding adequately treated basal cell or squamous cell skin cancer, or carcinoma *in situ*) within 3 years of enrollment; major surgery, receipt of other systemic anticancer therapy, and/or radiotherapy within 4 weeks before receiving PF-07209960; adverse events (AEs) from prior therapy not recovered to grade (G) ≤1 or baseline; history of G ≥3 immune-related AE considered related to prior immune modulatory therapy and required immunosuppressive therapy; active autoimmune disease that might deteriorate when receiving an immunostimulatory agent; current use of immunosuppressive medication; active bleeding disorder in the past 6 months; history of interstitial lung disease or pneumonitis; and were pregnant or breastfeeding.

### Objectives and endpoints

The primary objective of the dose-escalation part was to assess the safety and tolerability at increasing dose of PF-07209960 in patients with select locally advanced/metastatic solid tumors to estimate MTD and RP2D. The primary endpoints included DLTs, AEs, and laboratory abnormalities graded by the National Cancer Institute Common Terminology Criteria for Adverse Events version 5.0. The American Society for Transplantation and Cellular Therapy consensus criteria were used to grade the severity of cytokine release syndrome (CRS). Secondary objectives were to characterize the single and multiple dose PK of PF-07209960 and to evaluate the immunogenicity and preliminary antitumor activity of PF-07209960. The secondary endpoints were PK parameters; incidence, titers, and endogenous IL-15 cross-reactivity of anti-drug antibodies (ADA) and neutralizing antibodies (NAb) against PF-07209960; and objective response rate (ORR), disease control rate (DCR), duration of response (DoR), progression-free survival (PFS), and time to progression as assessed using RECIST version 1.1. Exploratory endpoints included overall survival (OS) and assessments of IL-15 signaling, PD-1 binding, PD-L1 expression, and other biomarkers.

#### Sampling for PK, pharmacodynamic, immunophenotyping, and cytokine activity

Serial blood PK samples were collected at pre-dose, 1, 4, 8, 24, 48, 72, and 168 h post-dose on cycle 1 day 1 (C1D1) and after multiple once-every-2-weeks (Q2W) doses on C2D15. Starting from cycle 3 onwards, pre-dose PK samples were collected on D1 of each cycle up to cycle 9 and then on D1 of every third cycle. A sample was also to be collected at the end of treatment (EOD)/withdrawal.

Paired tumor samples for immunohistochemistry (IHC) and RNAseq analyses were collected before and after treatment. IHC analysis was carried out at CellCarta (Montreal, Canada).

Blood samples for anti-PF-07209960 antibodies (ADA and NAb) were collected pre-dose on C1D1, C1D15, C2D1, and at the same time as the PK sampling starting from C3. Blood samples for pharmacodynamic, cytokine evaluation, and T-cell immunophenotyping were collected at pre-dose, 8, 24, 48, 72, and 168 h post-dose on C1D1; pre-dose on C1D15; pre-dose, 8, 24, 48, 72, and 168 h post-dose on C2D15; and pre-dose on C3D1. Blood samples were also collected at 8 h post-dose on C1D15 for cytokine evaluation, and at 4 h post-dose on C1D1 and C2D15 for pharmacodynamic assessment.

### Statistical analyses

There was no formal hypothesis testing in this study.

The starting dose was 1 mg subcutaneous injection Q2W. A two-parameter Bayesian logistic regression model guided by EWOC^26^ was used to guide the dose escalation. The DLT observation period was 28 days after C1D1. For MTD estimation, a minimum of six DLT-evaluable patients were required to be treated at that dose level.

Summaries were presented by dose group and overall. Descriptive statistics were provided for continuous endpoints. The rates of binary endpoints were provided with two-sided 95% confidence intervals (CIs) using an exact method. Time-to-event endpoints were summarized using the Kaplan–Meier method with median event times and two-sided 95% CIs.

## Results

### Patients

Thirty-seven patients were treated with PF-07209960 (1-, 3-, 10-mg groups, *n* = 4 each; 15 mg, *n* = 3; 20 mg, *n* = 16; 30 mg, *n* = 6). The median age was 59.0 years (range 31-88 years), 20 (54.1%) patients were male, 30 (81.1%) were White, and 27 (73.0%) were not Hispanic or Latino (Table 1). Primary diagnosis included CRC (*n* = 26), NSCLC (*n* = 1), OvCa (*n* = 4), and RCC (*n* = 6). The majority [20 (54.1%)] had ≥4 prior lines of anticancer drug therapy.

**Table 1:** Baseline characteristics

	PF-07209960 dose levels						Total (*N* = 37)
	1 mg (*n* = 4)	3 mg (*n* = 4)	10 mg (*n* = 4)	15 mg (*n* = 3)	20 mg (*n* = 16)	30 mg (*n* = 6)	
Age, years							
Median (range)	62.0 (56-88)	57.0 (46-82)	59.0 (54-71)	53.0 (50-59)	59.0 (36-74)	60.0 (31-67)	59.0 (31-88)
Mean (standard deviation)	67.0 (14.49)	60.5 (15.33)	60.8 (7.41)	54.0 (4.58)	56.6 (11.14)	54.8 (13.92)	58.1 (11.65)
Sex, *n* (%)							
Male	2 (50.0)	2 (50.0)	2 (50.0)	2 (66.7)	7 (43.8)	5 (83.3)	20 (54.1)
Race, *n* (%)							
White	3 (75.0)	3 (75.0)	4 (100.0)	3 (100.0)	12 (75.0)	5 (83.3)	30 (81.1)
Asian	0	0	0	0	3 (18.8)	0	3 (8.1)
Not reported	1 (25.0)	1 (25.0)	0	0	1 (6.3)	1 (16.7)	4 (10.8)
ECOG performance status, *n* (%)							
0	1 (25.0)	2 (50.0)	2 (50.0)	1 (33.3)	7 (43.8)	3 (50.0)	16 (43.2)
1	3 (75.0)	2 (50.0)	2 (50.0)	2 (66.7)	9 (56.3)	3 (50.0)	21 (56.8)
Follow-up anticancer drug therapy regimens							
0/Not reported	4 (100.0)	4 (100.0)	4 (100.0)	2 (66.7)	15 (93.8)	5 (83.3)	34 (91.9)
1	0	0	0	1 (33.3)	0	1 (16.7)	2 (5.4)
2	0	0	0	0	0	0	0
3	0	0	0	0	1 (6.3)	0	1 (2.7)

ECOG, Eastern Cooperative Oncology Group.

All patients discontinued treatment. The main reason for discontinuation was progressive disease [22 (59.5%) patients]; other reasons included patient’s decision [7 (18.9%)], physician’s decision [4 (10.8%)], health deterioration [2 (5.4%)], and death [2 (5.4%)].

The median duration of exposure was 1.41 months (range 0.0-13.6 months); for 23 (62.2%) patients, it was ≤3 months (Supplementary Table S1, available at https://doi.org/10.1016/j.esmoop.2025.104291). Dose reductions/interruptions due to all-causality AEs and treatment-related AEs (TRAEs) occurred in 20 (54.1%) and 12 (32.4%) patients, respectively. Five (13.5%) patients discontinued due to all-causality AEs; none of the AEs were considered treatment related.

### Safety

#### DLTs

Of the 27 patients evaluable for DLTs (i.e. had a DLT or had received both doses during cycle 1, and had all scheduled safety assessments during cycle 1), 6 (22.2%) had DLTs. Three patients received PF-07209960 20 mg, one each had resolved G3 CRS, G3 acute kidney injury, and G3 injection-site pain. The other three patients received PF-07209960 30 mg, one had fatigue [worsened from G2 (day 5) to G4 (day 8), all resolved], one had G3 rash (not resolved), and one had two episodes of G3 rash (the latter was not resolved) and a G3 mucosal inflammation (not resolved).

A G5 AE of anaphylactic reaction was assessed as not treatment related and not considered as DLT. Due to early termination, the MTD and RP2D were not established.

#### AEs

Overall, 36 (97.3%) patients had at least one all-causality AE, 23 (62.2%) had G3/4 AEs, 6 (16.2%) had G5 AEs, and 26 (70.3%) had serious AEs (SAEs).

Twenty-eight (75.7%) patients had at least one TRAE, 16 (43.2%) had G3/4 TRAEs, and none had G5 TRAEs. The most frequently reported TRAEs (≥50%) were general disorders and administration site condition [21 (56.8%)] and skin and subcutaneous tissue disorders [20 (54.1%)] (Table 2). The most frequently reported TRAE of any grade [17 (45.9%)] was CRS (Table 2), the majority was G1 (*n* = 10) and G2 (*n* = 6) with one G3, and the median time of CRS onset and resolution was 3.0 days (range 1.0-8.0 days) and 6.0 days (range 2.0-30.0 days), respectively (Supplementary Figure S2, available at https://doi.org/10.1016/j.esmoop.2025.104291). The most frequently reported G ≥3 TRAE was anemia [5 (13.5%)]. G4 TRAEs of fatigue, neutrophil count decreased, lymphocyte count decreased, and hyponatremia were reported in one patient each. Among patients treated with PF-07209960 20 mg, 8/16 (50%) had G ≥3 TRAEs, including acute kidney injury, dyspnea, lymphocyte count decreased (*n* = 2 each), and pyrexia (G3, *n* = 1; ungraded, *n* = 1) (Supplementary Table S2, available at https://doi.org/10.1016/j.esmoop.2025.104291).

**Table 2:** Most frequent TRAE in >15% of patients

	Grade 1	Grade 2	Grade 3	Grade 4	Total
With any adverse event	4 (10.8)	8 (21.6)	12 (32.4)	4 (10.8)	28 (75.7)
Cytokine release syndrome	10 (27.0)	6 (16.2)	1 (2.7)	0	17 (45.9)
Fatigue	1 (2.7)	9 (24.3)	1 (2.7)	1 (2.7)	12 (32.4)
Rash	4 (10.8)	6 (16.2)	2 (5.4)	0	12 (32.4)
Injection-site reaction	6 (16.2)	5 (13.5)	0	0	11 (29.7)
Nausea	8 (21.6)	1 (2.7)	0	0	9 (24.3)
Pyrexia	3 (8.1)	4 (10.8)	1 (2.7)	0	9 (24.3)^a^
Decreased appetite	4 (10.8)	3 (8.1)	0	0	7 (18.9)
Diarrhea	4 (10.8)	3 (8.1)	0	0	7 (18.9)
Alanine aminotransferase increased	4 (10.8)	2 (5.4)	0	0	6 (16.2)
Anemia	0	1 (2.7)	5 (13.5)	0	6 (16.2)
Aspartate aminotransferase increased	5 (13.5)	1 (2.7)	0	0	6 (16.2)
Chills	5 (13.5)	1 (2.7)	0	0	6 (16.2)
Dyspnea	3 (8.1)	1 (2.7)	2 (5.4)	0	6 (16.2)
Platelet count decreased	3 (8.1)	2 (5.4)	1 (2.7)	0	6 (16.2)
Pruritus	3 (8.1)	3 (8.1)	0	0	6 (16.2)

Values are *n* (%) by Preferred Term and maximum CTCAE grade. No grade 5 AE was reported. MedDRA v26.0 coding dictionary applied.

CTCAE, Common Terminology Criteria for Adverse Events; MedDRA, Medical Dictionary for Regulatory Activities; TRAE, treatment-related adverse events.

a Grade of one patient was unknown.

Treatment-related SAEs (TRSAEs) were reported in 13 (35.1%) patients. The most frequently reported TRSAEs (≥10%) were CRS [11 (29.7%)] and rash [4 (10.8%)]; the incidences of these TRSAEs were 7 (43.8%) and 2 (12.5%) for the PF-07209960 20-mg group.

#### Deaths

Seventeen (45.9%) patients died during the study; 8/37 (21.6%) during the treatment period due to disease under study (*n* = 5), unknown reason (*n* = 1), cardiac arrest/shock anaphylactic (*n* = 1), and large right retroperitoneal mass with internal hemorrhage/shock hemorrhagic (*n* = 1); 9 (24.3%) during the follow-up period due to disease under study (*n* = 1) and unknown reason (*n* = 8).

### Efficacy

**Figure 1:**
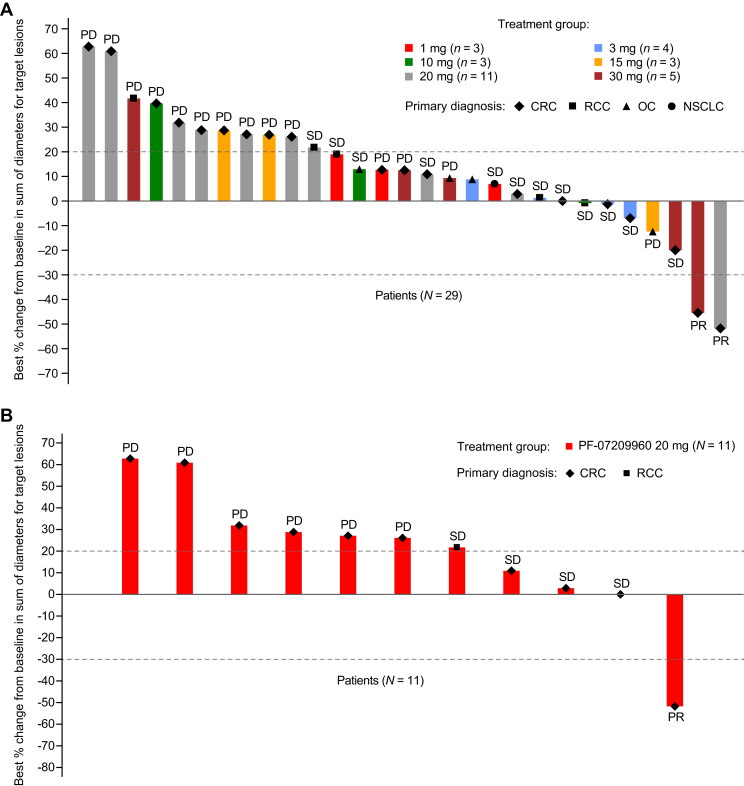

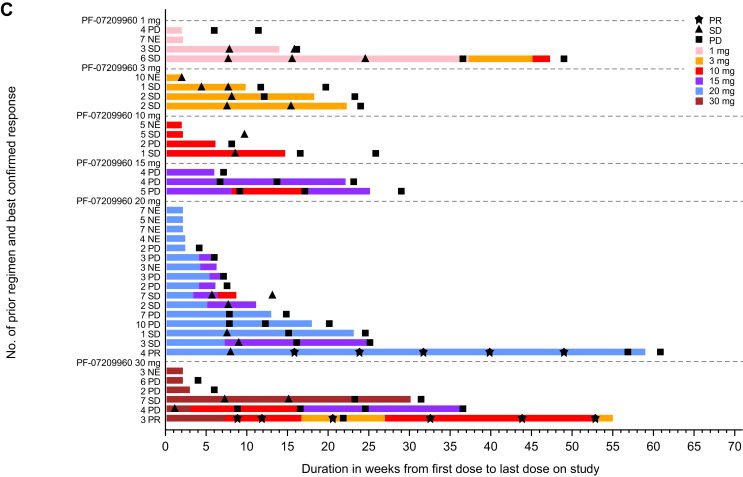
**Response and duration of treatment.** (A) Best percentage change in tumor size from baseline for the response-evaluable population. (B) Best percentage change in tumor size from baseline for patients treated with PF-07209960 20 mg who were evaluable for response. (C) Duration of treatment of the safety analysis population. CRC, colorectal cancer; NE, non-evaluable; NSCLC, non-small-cell lung cancer; OC, ovarian cancer; PD, progressive disease; PR, partial response; RCC, renal cell carcinoma; SD, stable disease.

There were 29 patients evaluable for response (i.e. patients who had measurable disease at baseline and had at least one post-treatment disease assessment). One patient from the 1-, 10-, and 30-mg cohort each and five from the 20-mg cohort were excluded due to lacking post-baseline disease assessment. Two patients (both with microsatellite-stable CRC) had confirmed partial response (PR), one each from the PF-07209960 20-mg and 30-mg cohorts (Table 3 and Figure 1), with a DoR of 9.5 and 3 months, respectively. The ORR was 6.9% (95% CI 0.8% to 22.8%) and the DCR [the percentage of patients with a best overall response of confirmed response (CR), PR, non-CR/non-progressive disease, or stable disease] was 48.3% (95% CI 29.4% to 67.5%) (Table 3 and Figure 1).

**Table 3:** Best overall response

	PF-07209960 dose levels						Total (*N* = 29)
	1 mg (*n* = 3)	3 mg (*n* = 4)	10 mg (*n* = 3)	15 mg (*n* = 3)	20 mg (*n* = 11)	30 mg (*n* = 5)	
Confirmed best overall response, *n* (%)							
Complete response (CR)	0	0	0	0	0	0	0
Partial response (PR)	0	0	0	0	1 (9.1)	1 (20.0)	2 (6.9)
Stable disease (SD)	2 (66.7)	3 (75.0)	2 (66.7)	0	4 (36.4)	1 (20.0)	12 (41.4)
Progressive disease (PD)	1 (33.3)	0	1 (33.3)	3 (100.0)	6 (54.5)	3 (60.0)	14 (48.3)
Non-CR/non-PD	0	0	0	0	0	0	0
Non-evaluable (NE)^a^	0	1 (25.0)	0	0	0	0	1 (3.4)
Objective response (CR + PR), *n* (%)	0	0	0	0	1 (9.1)	1 (20.0)	2 (6.9)
95% CI	0.0-70.8	0.0-60.2	0.0-70.8	0.0-70.8	0.2-41.3	0.5-71.6	0.8-22.8
Disease control							
CR + PR + SD + non-CR/non-PD, *n* (%)	2 (66.7)	3 (75.0)	2 (66.7)	0	5 (45.5)	2 (40.0)	14 (48.3)
95% CI	9.4-99.2	19.4-99.4	9.4-99.2	0.0-70.8	16.7-76.6	5.3-85.3	29.4-67.5

CI, confidence interval.

a Reason for NE was SD too early.

Twenty-eight of 37 (75.7%) patients had PFS events (progressive disease, *n* = 25; death, *n* = 3). The estimated probability of being progression free at 6 months was 8.0% (95% CI 1.4% to 22.3%). The Kaplan–Meier estimate of the median PFS was 2.0 months (95% CI 1.6-3.5 months) (Supplementary Table S3, available at https://doi.org/10.1016/j.esmoop.2025.104291). Among the 16 patients who received PF-07209960 20 mg, 11 (68.8%) had PFS events (progressive disease, *n* = 9; death, *n* = 2). The estimated probability of being progression free at 6 months was 11.1% (95% CI 0.7% to 37.8%). The Kaplan–Meier estimates of median PFS was 1.8 months (95% CI 1.4-3.5 months) (Supplementary Table S3 and Figure S3, available at https://doi.org/10.1016/j.esmoop.2025.104291).

Seventeen (45.9%) deaths were reported, and 20 (54.1%) patients were censored (withdrawal of consent, *n* = 11; alive, *n* = 8; lost to follow-up, *n* = 1). The estimated probability of survival at 12 months was 60.3% (95% CI 39.5% to 75.9%). The estimate of the median OS was 14.6 months (95% CI 7.9 months-non-evaluable) (Supplementary Table S4, available at https://doi.org/10.1016/j.esmoop.2025.104291). Among the 16 patients who received PF-07209960 20 mg, 5 deaths (31.3%) were reported and 11 (68.8%) were censored. The probability of survival at 12 months was 75.2% (95% CI 39.4% to 91.6%). The Kaplan–Meier estimates of median OS was not evaluable (Supplementary Table S4 and Figure S3, available at https://doi.org/10.1016/j.esmoop.2025.104291).

### PK

**Figure 2:**
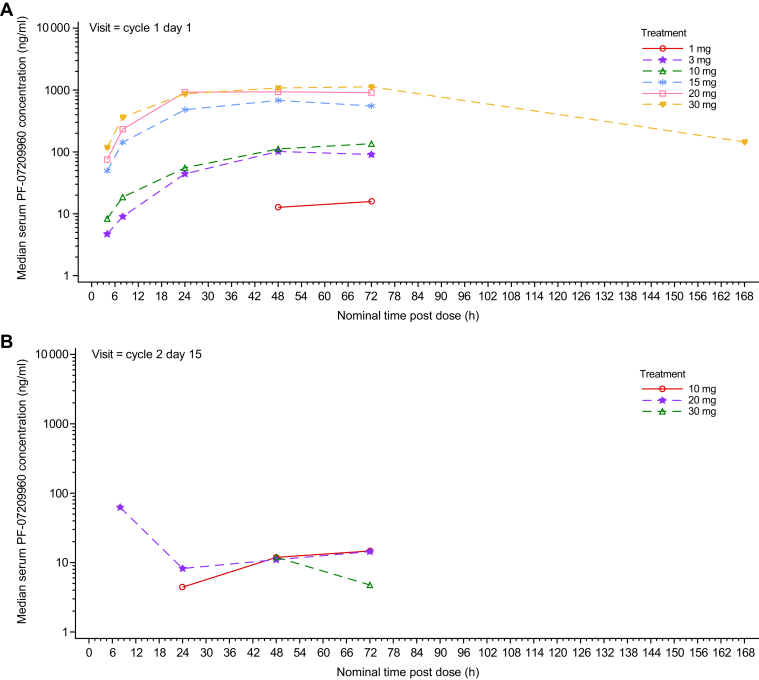
Median serum PF-07209960 concentration—PK analysis set.

Following a single dose of PF-07209960, most patients had measurable PK concentrations up to 72 h post-dose; of those who had available PK samples, 4/14 patients in the 20-mg cohort and 3/3 patients in the 30-mg cohort had measurable PK up to 168 h post-dose (Figure 2). Following multiple doses of PF-07209960 on C2D15, most patients showed PK concentrations as below limit of quantification (BLQ, <7.5 ng/ml) at all time points post-dose. For those who had measurable PK concentrations, the serum concentrations were markedly lower than those observed following the single dose, suggesting loss/reduction of serum exposure for PF-07209960 (Figure 2). All pre-dose serum PF-07209960 concentrations for C1D1 and beyond were BLQ in all patients.

### Immunogenicity

Overall, the incidence rate of ADA was 93.9% (31/33); 63.6% (21/33) was treatment induced whereas 30.3% (10/33) was treatment boosted (Supplementary Table S5, available at https://doi.org/10.1016/j.esmoop.2025.104291). The median time to detection of ADA across dose cohorts ranged from 14 to 24.5 days and aligned with the observed loss of PF-07209960 serum exposure with repeat dosing. The incidence of ADA specific against the PF-07209960 PD-1 domain and IL-15 mutein domain was 84.8% (28/33) and 66.7% (22/33), indicating that some patients had developed ADA against both domains of PF-07209960. The incidence of ADA against endogenous wild-type IL-15 was 30.0% (9/30). The incidence rate of NAb against endogenous wild-type IL-15 was 24.2% (8/33). The median time to first incidence of NAb ranged from 55 to 218 days across different dose cohorts.

### Pharmacodynamics

Pre-/post-treatment paired tumor biopsy samples (from liver) were collected from three patients. Two pairs were evaluable by IHC; one patient achieved PR and showed an increase of 36% (166.31-226.61 cells/mm^2^) and 344% (22.11-98.2 cells/mm^2^) in CD8+ and granzyme B+ cells, respectively, in the central tumor region; the other patient was non-responsive and showed an increase of 107% (123.13-255.1 cells/mm^2^) in CD8+ and 725% (10.97-90.5 cells/mm^2^) in granzyme B+ cells (Supplementary Figure S4A, available at https://doi.org/10.1016/j.esmoop.2025.104291). All three pairs were evaluable by RNAseq analysis. The PR patient showed a 769% (0.9062-7.8729 TPM) increase in *CD8* and a 59% (2.508-3.9884 TPM) increase in *granzyme B* mRNA; in addition, there were significant increases in *IFN-gamma*, *PD-L1*, *PD-1*, and *TBX21* mRNA. The other two patients also showed increase in the mRNAs of these genes (Supplementary Figure S4B, available at https://doi.org/10.1016/j.esmoop.2025.104291). The RNAseq results provided additional support of the CD8 and granzyme B IHC data and suggested that Th1 response might be enhanced in the post-treatment tumor microenvironment due to up-regulation of *IFNg* and *TBX21* gene expression.

Cytokine secretion before and after PF-07209960 treatment was assessed using the Olink proximity extension assay.^27^ IFN-gamma and CXCL10 were induced starting from 8 h and reached maximal induction by day 3-8 post-dose 1; limited data from post-dose 2 and 3 showed a gradual decrease in the extent of cytokine induction and only very limited induction by dose 4 (Supplementary Figure S5, available at https://doi.org/10.1016/j.esmoop.2025.104291), presumably due to the blocking effect of ADA. Some dose-dependency trend of cytokine induction was observed. IL-10, tumor necrosis factor-alpha, and CXCL9 also showed similar induction pattern. Overall, clear PD activities were identified in both tumor and peripheral blood after PF-07209960 treatment and the results suggest an increase of T-cell activity predominantly of a Th1 nature.

## Discussion

To optimally activate intratumoral CD8+ T cells enriched for the inhibitory marker PD-1, an anti-PD-1 antibody and IL-15 cytokine fusion approach was developed. Using this approach, an antibody–cytokine fusion molecule, PF-07209960, was designed to have decreased affinity for the IL-2/15 receptor to prevent systemic activation of NK cells and other PD-1-negative lymphocytes before reaching PD-1-expressing T cells. It was expected that for patients receiving PF-07209960, differences in peripheral versus intratumoral activation of PD-1-expressing T cells could allow systemically administered PF-07209960 to reach levels required for tumor-specific T-cell proliferation and cytotoxicity and may not necessarily lead to systemic toxicity.

Overall, PF-07209960 was generally tolerated. Four (10.8%) patients had dose interruption or reduction due to CRS, although all CRS events were resolved, and no patients discontinued the study due to CRS. Nevertheless, safety profile characterization over multiple cycles was not possible due to development of NAbs following cycle 1. Treatment with PF-07209960 resulted in rapid loss of serum exposure following repeated dosing, with the majority of patients showing BLQ levels after C2D15 dose. The loss of PF-07209960 exposure aligned with the development of ADA/NAb against PF-07209960. The observed incidence of treatment-induced ADA response to PF-07209960 treatment was high (93.9%). Treatment with PF-07209960 led to the development of ADA against both the PD-1 and IL-15 mutein domains of PF-07209960 and against endogenous wild-type IL-15. This ADA-mediated loss of exposure may have prevented full safety and tolerability assessment. Further, treatment-induced ADA may have an adverse effect on endogenous wild-type IL-15 and cause physiological dysfunctions that may impact cancer development. Nevertheless, two confirmed PRs were observed in patients with microsatellite-stable CRC. The patient who received PF-07209960 20 mg had a DoR of 9.5 months. This indicates potential benefit of PF-07209960 for some patients.

A multitude of clinical trials have been carried out to investigate recombinant IL-15 as a potential therapeutic agent as monotherapy or in combination with other agents in patients with various types of cancer.^28^, ^29^, ^30^, ^31^, ^32^ Findings of these studies would provide additional information on the approach of developing anti-PD-1/PD-L1 antibody-IL-15 as a cancer therapeutic agent. Other early-phase studies have suggested that recombinant IL-15 agonists in combination with immune checkpoint inhibitors (ICIs) may be beneficial for patients with cancers showing acquired resistance to ICIs.^33^, ^34^, ^35^, ^36^ Some IL-15 agonists are also being investigated in combination with other types of immunotherapies (e.g. cetuximab, Bacillus Calmette-Guérin).^37^, ^38^, ^39^ Besides various recombinant IL-15 proteins that are being investigated in clinical studies,^40^ new agents are being developed, such as immunocytokine LH01, an anti-PD-L1 antibody fused to IL-15 receptor alpha-sushi domain/IL-15 complex,^41^ and LH05, a tumor-conditional anti-PD-L1/IL-15 prodrug, which may be a promising candidate for treating patients with ICI resistance or cold tumors.^42^

As a phase I study, there are some limitations including a small sample size, which could limit the interpretation of the results. However, the safety and efficacy findings, together with evidence from other studies, indicate the potential benefit of recombinant IL-15 coupled with anti-PD-1/PD-L1 for some patients.

### Conclusions

Overall, PF-07209960 was generally tolerated, with clinical activity observed in two patients with microsatellite-stable CRC. The high incidence of immunogenicity against both the PD-1 and IL-15 mutein domain may have impacted the ability to fully access the longer-term clinical benefit and tolerability of PF-07209960.
