# Supplementary material for: First-in-human phase I study to evaluate safety, tolerability, pharmacokinetics, pharmacodynamics, immunogenicity, and antitumor activity of PF-07209960 in patients with advanced or metastatic solid tumors
Source: ESMO Open. 2025 Feb 17;10(3):104291. doi: 10.1016/j.esmoop.2025.104291 (PMC11876874; doi:10.1016/j.esmoop.2025.104291)
Supplement: Supplementary Figures and Tables [file mmc1.pdf]

Supplementary Materials

A Phase 1 Study to Evaluate Safety, Tolerability, Pharmacokinetics, Pharmacodynamics, and Anti-Tumor Activity of PF-07209960 in Patients With Advanced or Metastatic Solid Tumors

Aung Naing,[1] Meredith McKean,[2] Lee S Rosen,[3] David Sommerhalder,[4] Naveed Muzakkir Shaik,[5] I-Ming Wang,[5] Christophe Le Corre,[6] Kenneth A. Kern,[6] Natasha Homji Mishra,[7] Sumanta Kumar Pal[8]

[1]Department of Investigational Cancer Therapeutics, The University of Texas MD Anderson Cancer Center, Houston, TX, USA; [2]Sarah Cannon Research Institute (SCRI), Nashville, TN, USA; [3]UCLA Santa Monica Hematology-Oncology, Santa Monica, CA, USA; [4]Next Oncology, San Antonio, TX, USA; [5]Clinical Pharmacology and Translational Sciences, Pfizer Inc., La Jolla, CA, USA; [6]Pfizer, Inc., San Diego, CA, USA; [7]Pfizer Inc, San Francisco, CA, USA; [8]Department of Medical Oncology, City of Hope Comprehensive Cancer Center, Duarte, CA, USA.

Supplementary Table S1. Duration of treatment

	PF-07209960 dose levels						
	**1 mg (N=4)**	**3 mg (N=4)**	**10 mg (N=4)**	**15 mg (N=3)**	**20 mg (N=16)**	**30 mg (N=6)**	**Total (N=37)**
Duration of treatment, months							
Mean (standard deviation)	3.76 (4.915)	2.91 (2.257)	1.44 (1.371)	4.08 (2.368)	2.67 (3.417)	4.87 (5.245)	3.15 (3.555)
Median (range)	1.86 (0.5–10.9)	3.24 (0.0–5.1)	0.95 (0.5–3.4)	5.09 (1.4–5.8)	1.33 (0.0–13.6)	3.81 (0.0–12.6)	1.41 (0.0–13.6)
Category, months							
≤3	2 (50.0)	2 (50.0)	3 (75.0)	1 (33.3)	12 (75.0)	3 (50.0)	23 (62.2)
3-6	1 (25.0)	2 (50.0)	1 (25.0)	2 (66.7)	3 (18.8)	0	9 (24.3)
6-9	0	0	0	0	0	2 (33.3)	2 (5.4)
≥9	1 (25.0)	0	0	0	1 (6.3)	1 (16.7)	3 (8.1)

Supplementary Table S2. Most frequent TRAE in >15% of patients of the 20 mg group

	Grade 1	Grade 2	Grade 3	Grade 4	Total n (%)
With Any Adverse Event	3 (18.8)	3 (18.8)	6 (37.5)	2 (12.5)	14 (87.5)
Cytokine release syndrome	4 (25.0)	4 (25.0)	1 (6.3)	0	9 (56.3)
Pyrexia	3 (18.8)	2 (12.5)	1 (6.3)	0	7 (43.8)*
Nausea	6 (37.5)	0	0	0	6 (37.5)
Injection site reaction	2 (12.5)	3 (18.8)	0	0	5 (31.3)
Rash	2 (12.5)	3 (18.8)	0	0	5 (31.3)
Diarrhea	4 (25.0)	0	0	0	4 (25.0)
Fatigue	1 (6.3)	3 (18.8)	0	0	4 (25.0)
Pruritus	1 (6.3)	3 (18.8)	0	0	4 (25.0)
Alanine aminotransferase increased	2 (12.5)	1 (6.3)	0	0	3 (18.8)
Aspartate aminotransferase increased	2 (12.5)	1 (6.3)	0	0	3 (18.8)
Chills	3 (18.8)	0	0	0	3 (18.8)
Cough	3 (18.8)	0	0	0	3 (18.8)
Decreased appetite	1 (6.3)	2 (12.5)	0	0	3 (18.8)
Headache	3 (18.8)	0	0	0	3 (18.8)
Thrombocytopenia	1 (6.3)	1 (6.3)	1 (6.3)	0	3 (18.8)

Values are n (%) by Preferred Term and maximum CTCAE grade. No Grade 5 AE was reported. MedDRA v26.0 coding dictionary applied.

*Grade of 1 patient was unknown.

CTCAE, Common Terminology Criteria for Adverse Events; MedDRA, Medical Dictionary for Regulatory Activities TRAE, treatment-related adverse events.

Supplementary Table S3. Progression-free survival

	PF-07209960 dose levels						
	**1 mg (N=4)**	**3 mg (N=4)**	**10 mg (N=4)**	**15 mg (N=3)**	**20 mg (N=16)**	**30 mg (N=6)**	**Total (N=37)**
Patients with event, n (%)	4 (100.0)	3 (75.0)	2 (50.0)	3 (100.0)	11 (68.8)	5 (83.3)	28 (75.7)
Type of event, n (%)							
Progressive disease	3 (75.0)	3 (75.0)	2 (50.0)	3 (100.0)	9 (56.3)	5 (83.3)	25 (67.6)
Death	1 (25.0)	0	0	0	2 (12.5)	0	3 (8.1)
Patients censored, n (%)	0	1 (25.0)	2 (50.0)	0	5 (31.3)	1 (16.7)	9 (24.3)
Reason for censoring, n (%)							
No adequate baseline assessment	0	0	0	0	1 (6.3)	0	1 (2.7)
Withdrawal of consent	0	1 (25.0)	2 (50.0)	0	4 (25.0)	1 (16.7)	8 (21.6)
Probability of being event-free (95% CI)							
at 3 months	0.500 (0.058–0.845)	0.333 (0.009–0.774)	0.667 (0.054–0.945)	0.000 (NE–NE)	0.333 (0.103–0.588)	0.400 (0.052–0.753)	0.361 (0.195–0.530)
at 6 months	0.250 (0.009–0.665)	0.000 (NE–NE)	0.000 (NE–NE)	0.000 (NE–NE)	0.111 (0.007–0.378)	0.000 (NE–NE)	0.080 (0.014–0.223)
at 9 months	0.000 (NE–NE)	0.000 (NE–NE)	0.000 (NE–NE)	0.000 (NE–NE)	0.111 (0.007–0.378)	0.000 (NE–NE)	0.040 (0.003–0.169)
at 12 months	0.000 (NE–NE)	0.000 (NE–NE)	0.000 (NE–NE)	0.000 (NE–NE)	0.111 (0.007–0.378)	0.000 (NE–NE)	0.040 (0.003–0.169)
Kaplan-Meier estimates of time to event, months							
Quartiles (95% CI)							
Q1	1.6 (1.4–3.7)	2.7 (2.7–NE)	1.9 (1.9–NE)	1.5 (1.5–NE)	1.3 (1.0–1.8)	1.4 (0.9–5.0)	1.5 (1.3–1.8)
Median	2.8 (1.4–NE)	2.8 (2.7–NE)	3.8 (1.9–NE)	1.6 (1.5–NE)	1.8 (1.4–3.5)	2.0 (0.9–NE)	2.0 (1.6–3.5)
Q3	6.1 (1.9–NE)	5.5 (2.7–NE)	3.8 (1.9–NE)	2.1 (1.5–NE)	3.5 (1.7–NE)	5.0 (1.4–NE)	3.7 (2.7–5.4)

CI, confidence interval.

Supplementary Table S4. Overall survival

	PF-07209960 dose levels						
	**1 mg (N=4)**	**3 mg (N=4)**	**10 mg (N=4)**	**15 mg (N=3)**	**20 mg (N=16)**	**30 mg (N=6)**	**Total (N=37)**
Patients with event, n (%)	4 (100.0)	3 (75.0)	1 (25.0)	1 (33.3)	5 (31.3)	3 (50.0)	17 (45.9)
Patients censored, n (%)	0	1 (25.0)	3 (75.0)	2 (66.7)	11 (68.8)	3 (50.0)	20 (54.1)
Reason for censoring, n (%)							
Withdrawal of consent	0	1 (25.0)	2 (50.0)	0	6 (37.5)	2 (33.3)	11 (29.7)
Lost to follow-up [1]	0	0	0	1 (33.3)	0	0	1 (2.7)
Alive	0	0	1 (25.0)	1 (33.3)	5 (31.3)	1 (16.7)	8 (21.6)
Probability of being event-free (95% CI)							
at 3 months	0.750 (0.128–0.961)	1.000 (1.000–1.000)	1.000 (1.000–1.000)	0.667 (0.054–0.945)	0.859 (0.540–0.963)	0.800 (0.204–0.969)	0.835 (0.645–0.928)
at 6 months	0.500 (0.058–0.845)	0.333 (0.009–0.774)	0.500 (0.006–0.910)	0.667 (0.054–0.945)	0.859 (0.540–0.963)	0.800 (0.204–0.969)	0.683 (0.476–0.822)
at 9 months	0.250 (0.009–0.665)	0.333 (0.009–0.774)	0.500 (0.006–0.910)	0.667 (0.054–0.945)	0.752 (0.394–0.916)	0.800 (0.204–0.969)	0.603 (0.395–0.759)
at 12 months	0.250 (0.009–0.665)	0.333 (0.009–0.774)	0.500 (0.006–0.910)	0.667 (0.054–0.945)	0.752 (0.394–0.916)	0.800 (0.204–0.969)	0.603 (0.395–0.759)
Kaplan-Meier estimates of Time to Event, months							
Quartiles (95% CI)							
Q1	2.9 (1.9–7.9)	5.8 (5.8–NE)	3.6 (3.6–NE)	2.8 (2.8–NE)	14.0 (1.3–NE)	14.5 (2.9–17.0)	5.8 (2.9–14.0)
Median	5.9 (1.9–NE)	5.9 (5.8–NE)	NE (3.6–NE)	NE (2.8–NE)	NE (8.0–NE)	17.0 (2.9–NE)	14.6 (7.9–NE)
Q3	16.1 (3.9–NE)	16.7 (5.8–NE)	NE (3.6–NE)	NE (2.8–NE)	NE (14.6–NE)	NE (14.5–NE)	24.3 (16.7–NE)

CI, confidence interval.

Supplementary Table S5. Incidence of anti-PF-07209960 antibody (ADA) and anti-IL-15 wild type neutralizing antibody (NAb) - immunogenicity analysis set

	Total (N=37)				
	**ADA**	**ADA against PD-1**	**ADA against IL-15 mutein**	**ADA against endogenous IL-15 wild-type**	**NAb**
Total number of patients with ≥1 ADA or NAb result	37	35	35	32	35
ADA or Nab evaluable patients (N1)	33	33	33	30	33
Evaluable patients with pre-existing antibody, n/N1 (%)	12/33 (36.4)	1/33 (3.0)	8/33 (24.2)	1/30 (3.3)	0/33 (0.0)
Baseline-positive patients with non-boosted antibody response, n1/N1 (%)	2/33 (6.1)				
Overall incidence, n2/N1 (%)	31/33 (93.9)	28/33 (84.8)	22/33 (66.7)	9/30 (30.0)	8/33 (24.2)
Treatment-induced	21/33 (63.6)	28/33 (84.8)	22/33 (66.7)	9/30 (30.0)	8/33 (24.2)
Treatment-boosted	10/33 (30.3)				
Duration of ADA Response (for all ADA-positive patients)					
Transient	1/31 (3.2)	13/28 (46.4)	3/22 (13.6)	5/9 (55.6)	0/8 (0.0)
Persistent	11/31 (35.5)	4/28 (14.3)	6/22 (27.3)	0/9 (0.0)	3/8 (37.5)
Indeterminate	19/31 (61.3)	11/28 (39.3)	13/22 (59.1)	4/9 (44.4)	5/8 (62.5)

Baseline was defined as pre-dose measurement on Day 1.

A patient was ADA positive if the baseline titer was missing or negative and the patient had ≥1 post-treatment positive titer (treatment-induced), or with positive titer at baseline and a ratio of ≥4 in titer (dilution) to baseline in ≥1 post-treatment sample (treatment-boosted). Patients who were ADA positive at baseline but did not become boosted post-treatment were considered as ADA negative.

A patient was NAb positive if the baseline titer was missing or negative and the patient had ≥1 post-treatment positive titer (treatment-induced). NAb-negative patients included patients who were ADA negative or ADA-positive patients who tested negative in the NAb assay.

Transient: an ADA-positive or Nab-positive patient with (1) a treatment-induced or treatment-boosted ADA sample or treatment-induced NAb sample detected only at 1 sampling time (excluding the last time point) post-treatment, or (2) treatment-induced or treatment-boosted ADA samples or treatment-induced NAb detected at ≥2 time points where the first and last positive samples (irrespective of any negative samples in between) were separated by <16 weeks, and the patient's last sample was ADA negative or Nab negative.

Persistent: an ADA-positive or NAb-positive patient with first and last positive ADA samples (treatment-induced or treatment-boosted) or NAb samples (treatment-induced) detected over a period of ≥16 weeks post-treatment, irrespective of any negative samples in between.

Indeterminate: an ADA-positive or NAb-positive patient who was not persistent or transient.

N, number of patients in the treatment group; N1, number of patients with ≥1 post-treatment ADA or Nab result; n, number of ADA or Nab evaluable patients with positive ADA at baseline; n1, number of ADA evaluable patients with positive ADA at baseline but did not become boosted post-treatment; n2, number of ADA-positive or Nab-positive patients (treatment-induced or treatment-boosted; for Nab, only treatment-induced).

Supplementary Figure S1. Study design

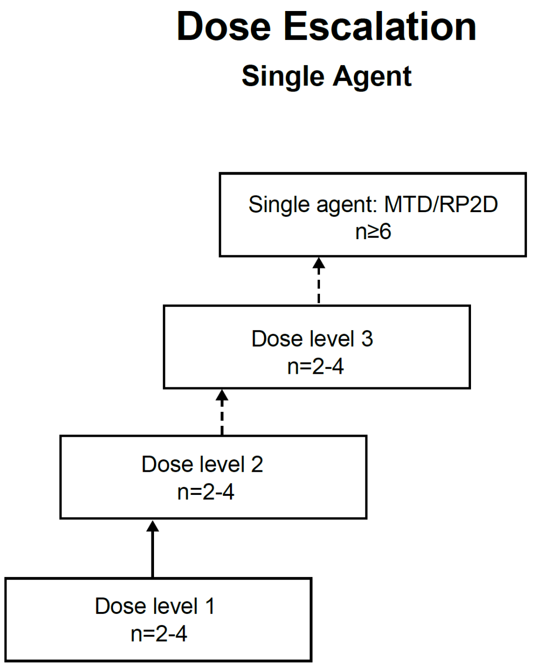

MTD, maximum tolerated dose; RP2D, recommended Phase 2 dose.

Supplementary Figure S2. All-causalities treatment-emergent adverse events: cytokine release syndrome

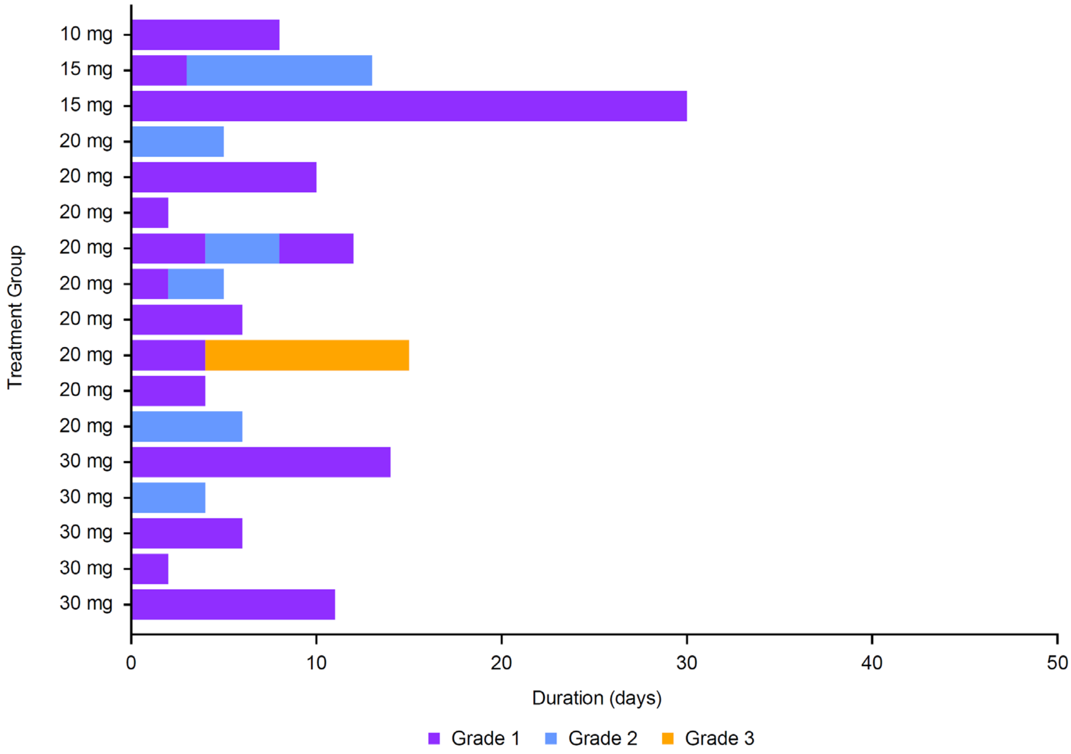

Supplementary Figure S3. Kaplan-Meier plot of progression-free survival and overall survival based on investigator assessment for patients treated at 20 mg - full analysis set

(A) Progression-free survival

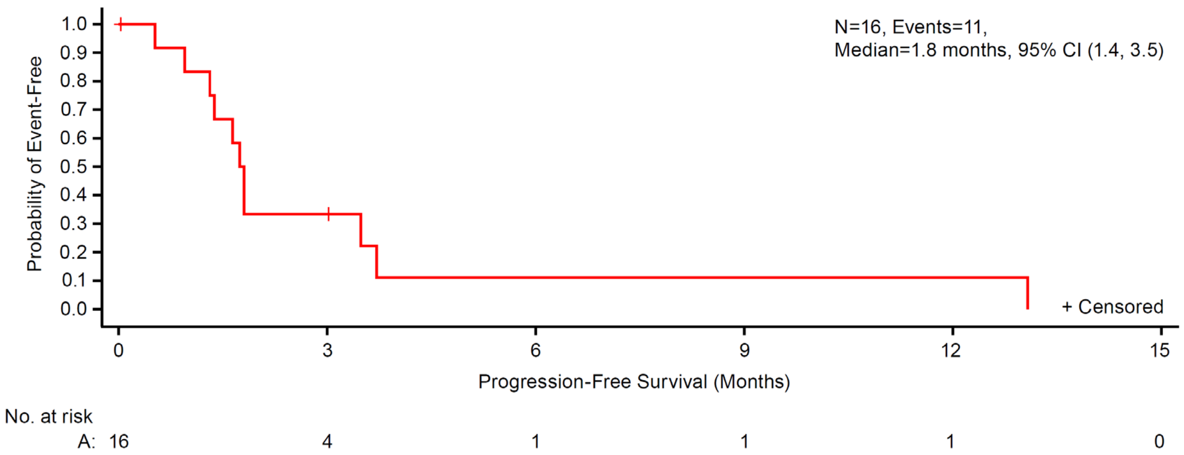

(B) Overall survival

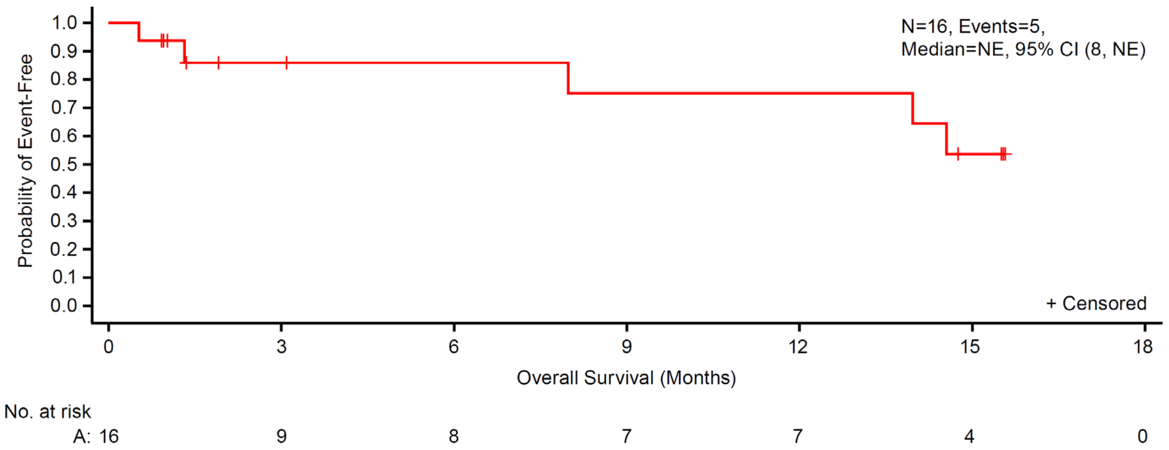

CI, confidence interval; NE, not evaluable.

Supplementary Figure S4. PF-07209960 increased effector CD8 T cell infiltration into tumor (A) and up-regulated Th1 related gene signature (B).

(A)

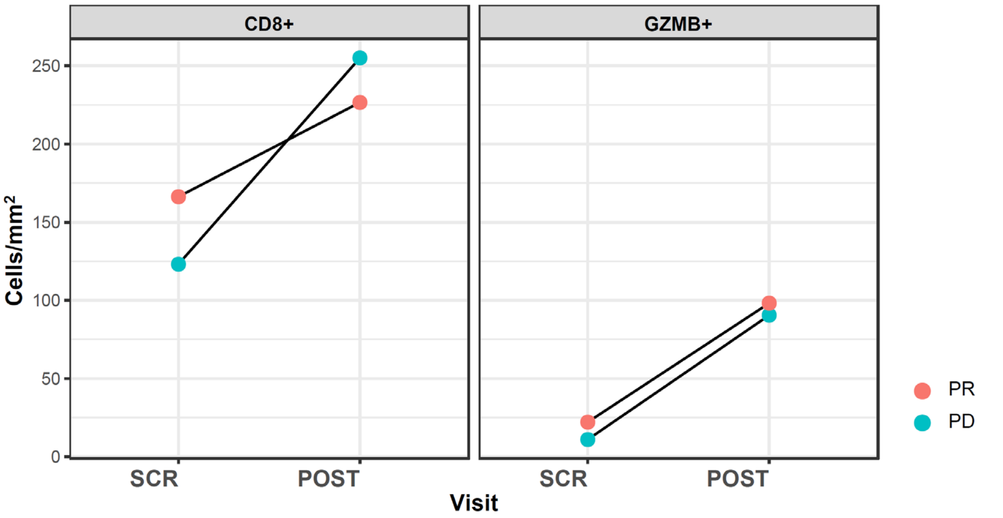

(B)

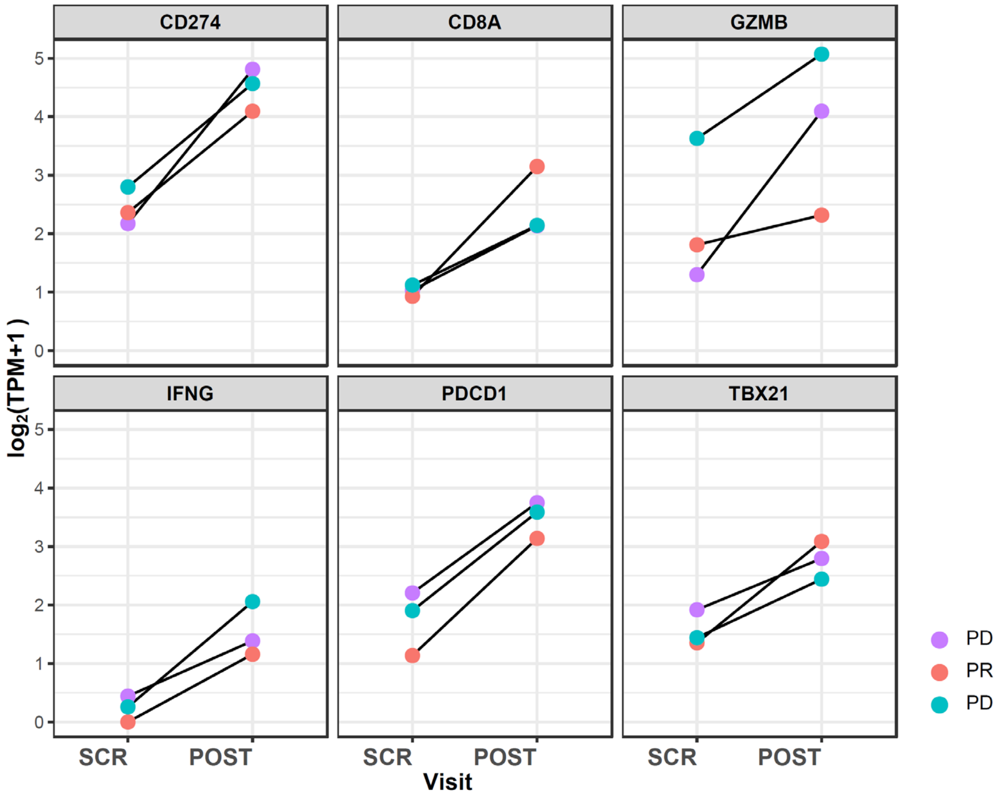

Samples were collected from the central tumor region in the liver.

CD, cluster of differentiation; GZMB, granzyme B; IFNG, Interferon gamma; IHC, immunohistochemistry; PD, progressive disease; PDCD1, programmed cell death protein 1; POST, post treatment; PR, partial response: SCR, screening (baseline); TBX21, T-box transcription factor 21; TPM, transcripts per million.

Supplementary Figure S5. PF-07209960 induced cytokine IFNG (A) and CXCL10 (B) post-dose 1-3, but induction level dropped gradually with very limited induction post-dose 4

(A)

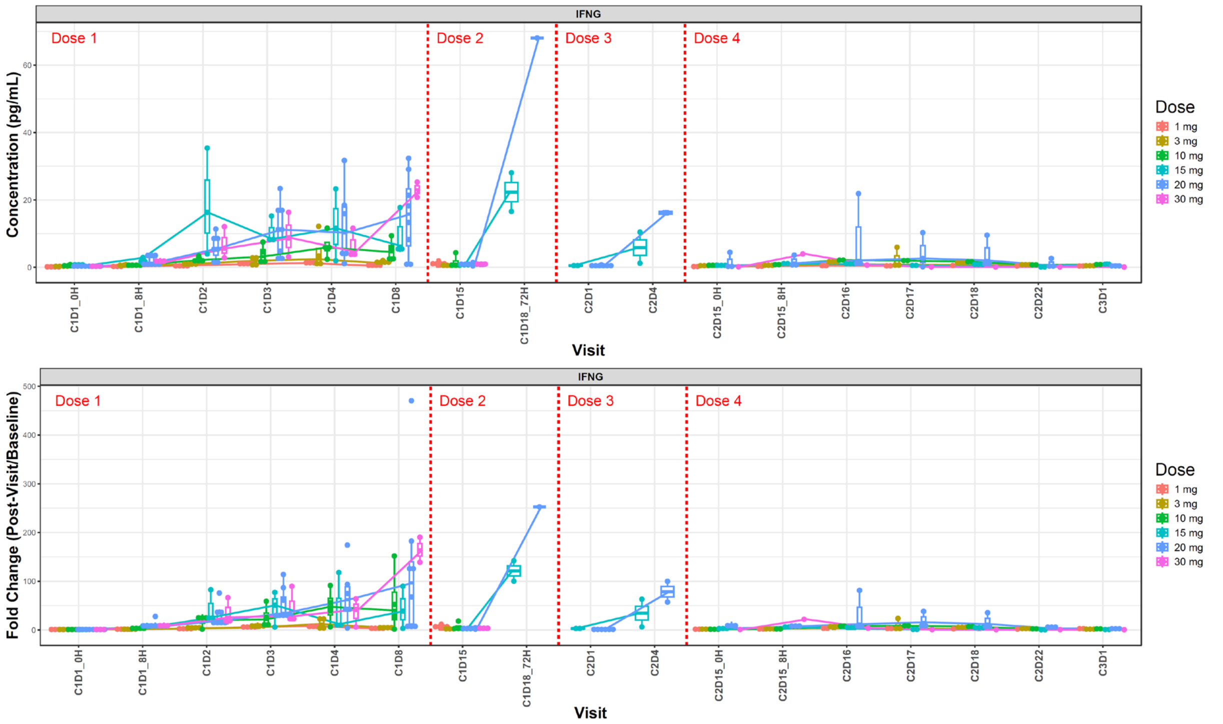

(B)

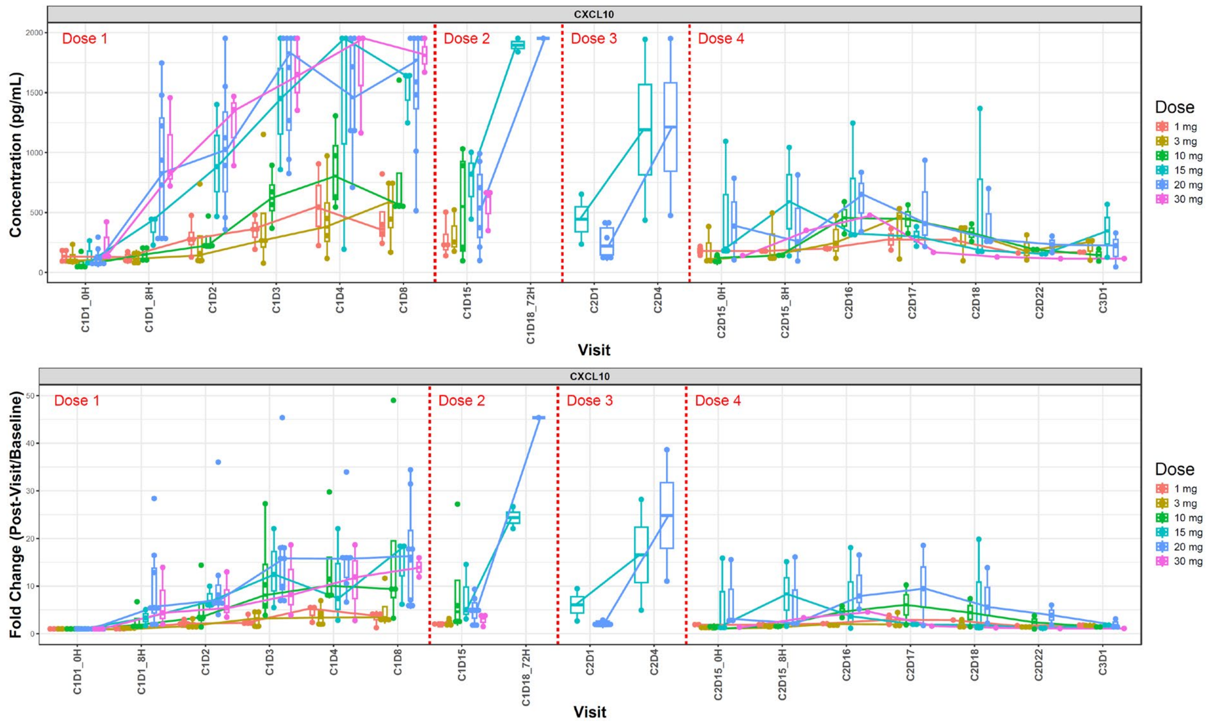

C, cycle; CXCL10, IFNG-inducible protein-10; D, day; IFNG, interferon gamma.
